# Uncontrollable Vomiting in Pregnancy

**Published:** 1898-12

**Authors:** John H. Hollister

**Affiliations:** Emeritus Professor Clinical Medicine Northwestern University School of Medicine, Consulting Physician at Mercer Hospital, Etc.


### UNCONTROLLABLE VOMITING IN PREGNANCY.

By JOHN H. HOLLISTER, A.M., M.D.

Emeritus Professor Clinical Medicine Northwestern University School of Medicine, Consulting Physician at Mercer Hospital, Etc.

Dr. Geoffroy (*Le Scalpel*, January 1, 1898) indicates a new method of treatment based on the employment of very gentle massage. This author, who has for a long time employed prolonged palpation in the treatment of affections of the digestive tract, has observed in several cases of uncontrollable vomiting that there existed at a point in the digestive tract a localized spasm transferred by reflex irritation from the uterus. This spasm, according to Dr. Geoffroy, was localized at two points: first, at the junction of the stomach with the duodenum; second, at the point well down in the left iliac fossa, at the posterior part of this fossa at a point which is never any other than the ileo-pelvic angle of the colon, formed by the union of the fixed iliac part of the colon with the movable pelvic part. This spasm causes a permanent state of contraction, which deep palpation readily discloses. This peculiarity is for the writer so characteristic that when he encounters it in a woman who vomits or has a cessation of the menses he diagnoses pregnancy. This contraction is encountered in subjects affected with various gastro-intestinal troubles, neurasthenia, the beginning of appendicitis, etc. It is a pathognomonic sign of reflex hyperesthesia of the intestinal canal, of which the morbid symptoms vary from those produced by mild cardiac disturbances and simple nausea to uncontrollable vomiting. By using precautions to avoid pain in making gentle pressure, both slow and progressive, with the ball and not with the extremity of the fingers, one can perceive at the point mentioned a series of nodules, ordinarily very small, which are hard and resisting, but which become softened in proportion as the digital pressure is gentle, slow and progressive. There is a sensation of gas and of liquids under the fingers, with a slight gurgling, generally perceptible to both the physician and the patient. The induration ceases, the nodules disappear, and the discomfort that they had excited is replaced by a sensation of well being and an inclination to rest, which indicate that the time has come to suspend the treatment. In the first case in which the author applied this treatment, five or six seances were requisite to effect a complete cure. In general, two or three seances, sometimes only one, cause the vomiting to cease. It is useless to prolong the treatment in order to avoid a return of the trouble The method is useful in all cases if properly applied, and is perfectly harmless.—*Medical Record*.

